# Engagement of Husbands in a Maternal Nutrition Program Substantially Contributed to Greater Intake of Micronutrient Supplements and Dietary Diversity during Pregnancy: Results of a Cluster-Randomized Program Evaluation in Bangladesh

**DOI:** 10.1093/jn/nxy090

**Published:** 2018-06-20

**Authors:** Phuong Hong Nguyen, Edward A Frongillo, Tina Sanghvi, Gargi Wable, Zeba Mahmud, Lan Mai Tran, Bachera Aktar, Kaosar Afsana, Silvia Alayon, Marie T Ruel, Purnima Menon

**Affiliations:** 1 Poverty, Health, and Nutrition Division, International Food Policy Research Institute, Washington, DC; 2 University of South Carolina, Columbia, SC; 3 FHI 360, Washington, DC; 4 Division of Nutritional Sciences, Cornell University, Ithaca, NY; 5 James P Grant School of Public Health, BRAC University, Dhaka, Bangladesh; 6 BRAC, Dhaka, Bangladesh

**Keywords:** Bangladesh, cluster-randomized trial, interpersonal counseling, engagement of husbands, maternal nutrition program

## Abstract

**Background:**

Although husbands may provide support during pregnancy, limited evidence exists on how to promote husbands’ engagement and what impact it has. Alive & Thrive integrated nutrition-focused interventions, targeting both wives and husbands, through an existing Maternal, Neonatal, and Child Health (MNCH) platform in Bangladesh.

**Objectives:**

We evaluated *1*) the impact of a nutrition-focused MNCH program, compared with the standard MNCH program, on husbands’ behavioral determinants (i.e., awareness, knowledge, self-efficacy) and support to wives to adopt optimal nutrition practices and *2*) how much of the previously documented impact on women's supplement intake and dietary diversity was explained by husbands’ behavioral determinants and support.

**Methods:**

We used a cluster-randomized design with cross-sectional surveys at baseline (2015) and endline (2016) (*n* = ∼1000 women and ∼700 husbands/survey). We used mixed linear regression accounting for clustering to estimate difference-in-differences (DIDs) for impact on husbands’ behavioral determinants and path analysis to examine how much these determinants explained the impact on women's nutrition behaviors.

**Results:**

Of husbands in the nutrition-focused MNCH group, 62% were counseled by health workers, 66% attended a husbands’ forum, and 34% saw video shows. The nutrition-focused MNCH, compared with the standard MNCH group, resulted in greater husbands’ awareness (DID: 2.74 of 10 points), knowledge (DID: 1.31), self-efficacy and social norms with regard to optimal nutrition practices (difference: 1.08), and support to their wives (DID: 1.86). Husbands’ behavioral determinants and support explained nearly half of the program impact for maternal supplement intake and one-quarter for dietary diversity.

**Conclusions:**

A nutrition-focused MNCH program that promoted and facilitated husbands’ engagement during their wives’ pregnancies significantly improved husbands’ awareness, knowledge, self-efficacy, and support. These improvements substantially explained the program's impact on women's intake of micronutrient supplements and dietary diversity. Targeting wives and husbands and designing activities to engage men in maternal nutrition programs are important to maximize impact. This trial was registered at www.clinicaltrials.gov as NCT02745249.

## Introduction

Undernutrition among women of reproductive age, manifested by short stature, underweight, and micronutrient deficiencies, remains pervasive in Asia and Africa (1). Nutritional deficit during pregnancy contributes to maternal deaths during childbirth (1, 2), adverse birth outcomes (3), and stunting by age 2 y (4). Maternal undernutrition also contributes to deaths and preventable illnesses in >1,000,000 children before age 5 y (2). Thus, improving maternal nutrition during pregnancy is important to reduce the global burden of maternal and child undernutrition, morbidity, and mortality (5).

The WHO recently released new recommendations on antenatal care (ANC), which include specific recommendations to improve diets and nutrient intake during pregnancy, along with health assessments, disease prevention, and health system strengthening to improve the utilization and quality of ANC (6). The guidelines recommend that, in addition to receiving specific micronutrient supplements and balanced energy and protein dietary supplementation (in food-insecure areas), women should receive relevant and timely counseling about healthy diets, physical activity, and adequate weight gain during pregnancy. To date, most large-scale nutrition interventions in developing countries either focus on specific micronutrient supplementation (7–11) or balanced energy and protein supplementation (3, 12) and solely target women of childbearing age without regard for their autonomy within the household and their ability to adopt the promoted behaviors on their own (13, 14). Engaging husbands, especially when they are the head of the household and primary decision makers on family health care, is important to improve care-seeking for women and children (15, 16).

The importance of male partner support for improving maternal health-seeking behaviors and practices is evident in the health literature related to the prevention of mother-to-child transmission of HIV (17), family planning (18), maternal care (19), and breastfeeding (20). Studies in Peru (21), Zimbabwe (22), and Kenya (23) have also highlighted the importance of spousal support to improve adherence to micronutrient supplements among pregnant women. In addition, fathers’ support is reported to avert postpartum maternal depression, which, in turn, affects the mother-child interaction during breastfeeding and child feeding (24). Although these studies underscore the benefits of engaging husbands for specific nutritional interventions, evidence of the impact of interventions targeting husbands to improve maternal nutrition is lacking. Including husbands in social behavior-change programs for nutrition is particularly important in South Asia, where the prevalence of maternal short stature and anemia remains critically high (1, 2), women's decision-making autonomy is particularly low, and households are predominantly male-headed (25, 26).

In Bangladesh, more than half of pregnant women are anemic, and 20% of ever-married women 15–49 y of age are underweight (27, 28). Nutritional inadequacies among women and children are high due to a lack of dietary diversity and low intake of micronutrient supplements (29). Yet, less than half of pregnant women received iron and folic acid (IFA) during antenatal consultations (30), and women often consumed only ≤90 IFA tablets (31), an amount that is lower than recommended.

To address the challenges of maternal undernutrition in Bangladesh, the Alive & Thrive initiative leveraged BRAC’s existing Maternal, Neonatal, and Child Health (MNCH) platform (i.e., “standard MNCH” program) and incorporated a strong maternal nutrition package of interventions to *1*) promote increased dietary intake, diet diversity, and adequate rest; *2*) provide free IFA and calcium supplements; and *3*) monitor weight gain during pregnancy. This integrated program is referred to as the “nutrition-focused MNCH” program. We showed previously that, compared with the standard MNCH program, the nutrition-focused MNCH program successfully improved multiple outcomes such as maternal dietary diversity and micronutrient supplement consumption during pregnancy and exclusive breastfeeding practices (32). Given the recognized importance of male partner support for improving maternal health-seeking behaviors and practices (15, 19), the nutrition-focused MNCH also included specific interventions to promote greater engagement of husbands and support to their wives during pregnancy. In this article, we report results from our analysis of the following: *1*) the impact of providing nutrition-focused MNCH compared with standard MNCH programs on husbands’ awareness, knowledge, self-efficacy, and support for optimal nutrition practices of their wives and *2*) the extent to which the impact of the program previously reported on micronutrient supplement intake and dietary diversity was explained by differences in the husbands’ engagement in the program as reflected by differences in related behavioral determinants and support to their wives during pregnancy.

## Methods

### 

#### Study context and intervention description

This study used data from a cluster-randomized study conducted to evaluate the feasibility and impact of integrating intensified maternal nutrition interventions into the existing MNCH program platform in Bangladesh. A detailed description of the intervention package has been provided elsewhere (32). Briefly, Alive & Thrive designed a nutrition-focused MNCH program (including interpersonal counseling, community mobilization, distribution of free micronutrient supplements, and weight-gain monitoring) targeted to both wives and husbands, with the overall goal of improving maternal nutrition. All components of the maternal nutrition interventions started in 10 subdistricts in August 2015 and continued until the end of August 2016.

Interpersonal counseling was delivered by 2 frontline workers, Shasthya Kormi (SK; salaried health worker) and Shasthya Shebika (SS; community health volunteer worker), through monthly home visits for all pregnant women. Husbands of these women were encouraged to attend the home-based counseling sessions as well. The nutrition-focused interventions delivered during home visits included the following: *1*) demonstrating a specific diet plan (both quality and quantity), *2*) providing free supplements (IFA and calcium tablets) and advice on using them, *3*) measuring weight and explaining optimal weight-gain patterns, *4*) counseling on adequate rest during pregnancy, and *5*) engaging husbands and other family members to ensure enough varied foods and supplements being available and supporting pregnant women to consume them.

Community mobilization in the nutrition-focused MNCH model involved husbands’ forums (i.e., meetings), small group meetings with community opinion leaders, entertaining video shows, and popular theater for the entire community. Husbands of pregnant women were invited to attend 2 husbands’ forums during pregnancy (in the second and third trimesters) to discuss several topics including the following: *1*) benefits of ensuring good maternal nutrition for the mother and child, *2*) husbands’ role in purchasing nutritious foods and motivating their mothers and wives to ensure that the recommended quantities of diverse foods are consumed by their wives daily, *3*) ensuring adequate supplies of IFA and calcium supplements and reminding their wives to take them daily, and *4*) reviewing their wives’ weight gain and supporting healthy weight gain. Video shows and interactive communication were carried out for the community, covering multiple topics related to nutrition during pregnancy and aiming at shifting social norms through spreading awareness about the recommended practices and the need to support pregnant women. These targeted multiple audiences included women, their husbands and family members, local elites (religious leaders, community leaders, teachers), village doctors, medical drug sellers, pharmacists, and government health workers. In the standard MNCH program, women received ANC with standard nutrition counseling; there were no community mobilization or husband engagement activities being promoted.

#### Study design and participants

The study design has been described in detail elsewhere (32). Briefly, a cluster-randomized, nonblinded, impact-evaluation design was used to compare the nutrition-focused MNCH and the standard MNCH programs. Twenty subdistricts (*upazilas*) from 4 districts (Mymensingh, Rangpur, Kurigram, and Lalmonirhat), where BRAC's rural MNCH program already existed, were randomly assigned to nutrition-focused MNCH or standard MNCH. Cross-sectional household surveys were conducted at baseline (July–August 2015) and endline (July–August 2016) in the same villages.

A total of 1000 women with children aged <6 mo and ∼70% of their husbands (except for those who worked far from home) were surveyed in each group and at each survey round. To obtain the samples, within each subdistrict, 5 unions and 2 villages within each union were randomly selected to yield a total of 200 villages (average size of 250 households/village). Within each village, a household census was conducted before each round to create a list of mothers with infants <6 mo of age. Households were selected for surveys by using systematic sampling beginning with a random seed starting point to yield the desired sample size per cluster. Data were collected via face-to-face interviews with women and their husbands in a separate room in the household with the use of a structured questionnaire, which was prepared in English, translated into Bangla, and then back-translated. Enumerators were trained by mixed methods (lecture, role-play, mock interview, and practice) in classroom and field settings.

#### Measurements

Husbands’ exposure to program interventions was assessed by 3 indicators: receiving advice related to maternal nutrition from frontline workers during home visits, attending a nutrition-related husbands’ forum, and participating in interactive video shows in the community. The husbands’ behavioral determinants measured included awareness of the recommended maternal nutrition practices, knowledge of the benefits and practices, and self-efficacy and perceptions of social norms related to maternal nutrition. Support provided by the husband to his wife during pregnancy was measured by recall from their wives. On the basis of the Theory of Reasoned Action (33), an integrative framework for the prediction of and change in human social behavior, we postulated that a husband's exposure to the program would lead to a change in his awareness, knowledge, and self-efficacy and perception of social norms. These determinants, in turn, would result in husbands taking supportive actions to support their pregnant wives in taking IFA and calcium supplements as recommended, and improving their dietary diversity.

To measure awareness, husbands were asked if they had ever heard any of several messages related to proper diet during pregnancy, intake of IFA and calcium, optimal weight gain, and the need to rest during pregnancy. Each awareness item was given a score of 1 (yes) and 0 (no) and the sum was used as the awareness score. The husband's knowledge was assessed by asking a list of open-ended questions about IFA and calcium (benefit of taking IFA or calcium during pregnancy, how long women should take IFA and calcium during pregnancy, and knowledge of the foods that interfere with IFA absorption) and types of food that pregnant women should eat every day. Each knowledge item was given a score of 1 (correct) or 0 (incorrect), and the sum was used as the knowledge score. Items to measure the husband's self-efficacy (his confidence in being able to provide the expected support to his wife) and perceptions of social norms (his perceptions about community practices) were combined into a single score due to a high correlation with each other. The items included statements with regard to the husband's confidence in his capacity to provide sufficient food and supplements for his pregnant wife, his commitment to remind his wife to take supplements daily, and his beliefs that most other husbands in the village similarly support their wives. Responses to each statement were made on a 5-point Likert scale in which husbands reported the degree to which they agreed or disagreed. Responses to negative statements were reverse coded, so all responses were in the same direction as positive items. Cognitive and pilot tests were conducted for all items to ensure that husbands understood their meaning. Each item was given a score of 1 (agree) or 0 (disagree), and the sum was used as the score for self-efficacy and perceptions of social norms. Finally, the husband's support was measured by asking his wife if she received different forms of support from him during pregnancy, including whether he purchased diversified nutritious foods, ensured adequate supplies of IFA and calcium, and reminded her and supported her to practice the recommended behaviors. The total scores for each type of husband's behavioral determinants were standardized to the theoretical range of each scale from 0 to 10 for comparability.

Covariates potentially associated with uptake of interventions were also measured. These included age, education, and occupation for both women and their husbands and household size, number of children aged <5 y, and socioeconomic status at the household level. The measure of socioeconomic status was obtained by using principal components analysis for a set of items related to household assets, housing conditions, and access to utilities (34).

The consumption of IFA and calcium supplements was assessed by asking women to report how many IFA or calcium tablets they consumed during their last pregnancy. During monthly visits to women's homes, frontline workers recorded the number of IFA and calcium tablets consumed in a mother-child handbook as part of the MNCH program; this book was used to assist women in their recall. Maternal dietary diversity was assessed by using a quantitative 24-h recall, in which all foods consumed were categorized in 10 food groups to create the Women's Dietary Diversity Score (35). The dietary diversity measure represents the number of food groups consumed and thus ranged from 0 to 10.

#### Statistical analysis

The differences between the 2 study groups, standard MNCH and nutrition-focused MNCH, in each survey round were tested with the use of linear (continuous variables) or logit (categorical variables) regression models for each item of the behavioral determinants. We used mixed, 3-level regression models that estimated the difference between the 2 study groups in the differences over time [difference-in-difference (DID)] as the interaction between survey round and study group for the overall score of each behavioral determinant (36). All of the models accounted for clustering at the subdistrict and village levels by including these as random effects (37) and adjusted for household socioeconomic status and husbands’ and wives’ education that were different at endline. Path analysis (38) was used to examine how much of the program differences in intake of IFA supplements, calcium supplements, and dietary diversity were explained by husbands’ behavioral determinants. The path models used endline data, and the indirect association for each outcome indicator was calculated as the product of the unstandardized regression coefficients for each path. Data analysis was performed with the use of Stata 14 (StataCorp).

#### Ethical approval

Approval was obtained from the institutional review boards at the International Food Policy Research Institute and the Bangladesh Medical Research Council. All women were provided with detailed information in writing and verbally at recruitment. Written informed consent was obtained from all women aged >18 y. For women aged <18 y, we obtained their assent and the permission from their guardians. The study was registered at www.clinicaltrials.gov as NCT02745249.

## Results

### 

#### Sample characteristics

On average, husbands were 31 y old; nearly one-quarter had no formal education and >40% worked as farmers. The mean age of their wives was 24 y (**Table 1**). The majority of women were housewives with an average of 6 y of education. More than 10% of women had no schooling and >80% did not complete high school. There were no differences in characteristics of wives, husbands, and households between nutrition-focused MNCH and standard MNCH areas at baseline, indicating that randomization was successful. At endline, husbands and wives in the nutrition-focused MNCH had a slightly higher educational level, and households had higher socioeconomic status, compared with the standard MNCH group.

**TABLE 1:** Selected characteristics of husbands and their wives at baseline and endline^1^

	Baseline		Endline	
	Nutrition-focused	Standard	Nutrition-focused	Standard
Characteristics	MNCH	MNCH	MNCH	MNCH
Husbands, *n*	747	767	622	685
Age, y	31.6 ± 7.2	31.2 ± 7.1	32.1 ± 6.9	31.7 ± 6.9
Occupation, %				
Farmer	43.5	46.3	40.4	44.2
Worker	19.4	15.6	13.5	14.7
Traders	21.0	18.1	23.6	16.5
Rickshaw/van pulling/self-employment/others	16.1	20.0	22.5	24.5
Education, %				
No schooling	26.7	24.0	21.9	21.2
Primary school	38.0	37.8	34.9*	41.8
Secondary school	22.4	23.7	27.2	21.9
High school, college or higher	12.9	14.5	16.1	15.2
Women, *n*	1000	1000	1000	1000
Age, y	24.7 ± 5.43	24.2 ± 5.58	24.8 ± 5.40	25.1 ± 5.61
Age at marriage, y	16.7 ± 2.4	16.5 ± 2.3	16.7 ± 2.4	16.9 ± 2.5
Occupation as housewife, %	89.4	90.3	96.4	95.0
Education, %				
No schooling	10.7	12.8	9.00^†^	12.0
Primary school	36.4	33.9	31.1	33.5
Secondary school	37.9	37.9	42.9^†^	39.6
High school, college or higher	15.0	15.4	17.0	14.9
Households, *n*	1000	1000	1000	1000
Household size, *n*	5.2 ± 1.9	5.0 ± 1.8	5.2 ± 1.9	5.0 ± 1.6
Number of children <5 y of age	1.3 ± 0.5	1.3 ± 0.5	1.3 ± 0.5	1.2 ± 0.4
Socioeconomic index^2^	–0.06 ± 0.99	–0.06 ± 0.96	0.15 ± 0.98*	–0.03 ± 0.84

^1^Values are means ± SDs unless otherwise indicated. ^†^*P* < 0.10, *P < 0.05. MNCH, Maternal, Neonatal, and Child Health.

^2^The socioeconomic index was constructed by using principal components analysis with variables on ownerships and assets. It is a standardized score with mean = 0 and SD = 1.

#### Husbands’ exposure to program intervention

**FIGURE 1:**
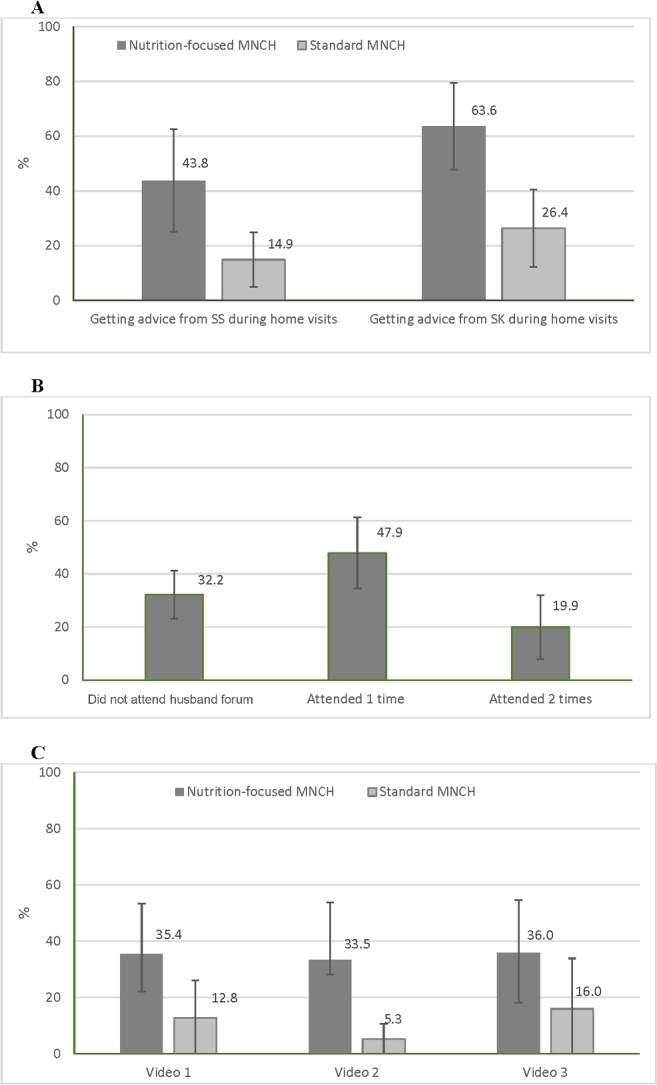
Husbands’ exposure to program interventions at endline showing exposure to counseling from frontline workers’ home visits (A), husbands’ forum (B), and video shows (C). Intervals shown are ±1 SD among subdistricts. Video 1: “Everyone has a responsibility to ensure nutrition and care for pregnant women”; Video 2: “Five rules of pregnant women's nutrition during pregnancy”; Video 3: “Nutritious foods are easily available.” MNCH, Maternal, Neonatal, and Child Health; SK, Shasthya Kormi (salaried health worker); SS, Shasthya Shebika (community health volunteer worker).

The percentage of husbands who received advice related to maternal nutrition from frontline workers was higher in nutrition-focused MNCH than in standard MNCH areas at endline (62% compared with 27% for SK and 41% compared with 16% for SS) (**Figure 1**). Two-thirds of husbands in the nutrition-focused MNCH areas reported ever attending a husbands’ forum, and most of them attended while their wives were in the second trimester of pregnancy. In the nutrition-focused MNCH areas, approximately one-third of husbands reported ever having seen video shows in the community, which was significantly higher than in the standard MNCH areas. Husbands’ exposure to program components varied across subdistricts. For example, exposure to SK, SS, husbands’ forum, and video shows ranged from 37% to 83%, 10% to 80%, 52% to 80%, and 6% to 77%, respectively, across subdistricts.

#### Husbands’ awareness related to maternal nutrition

**FIGURE 2:**
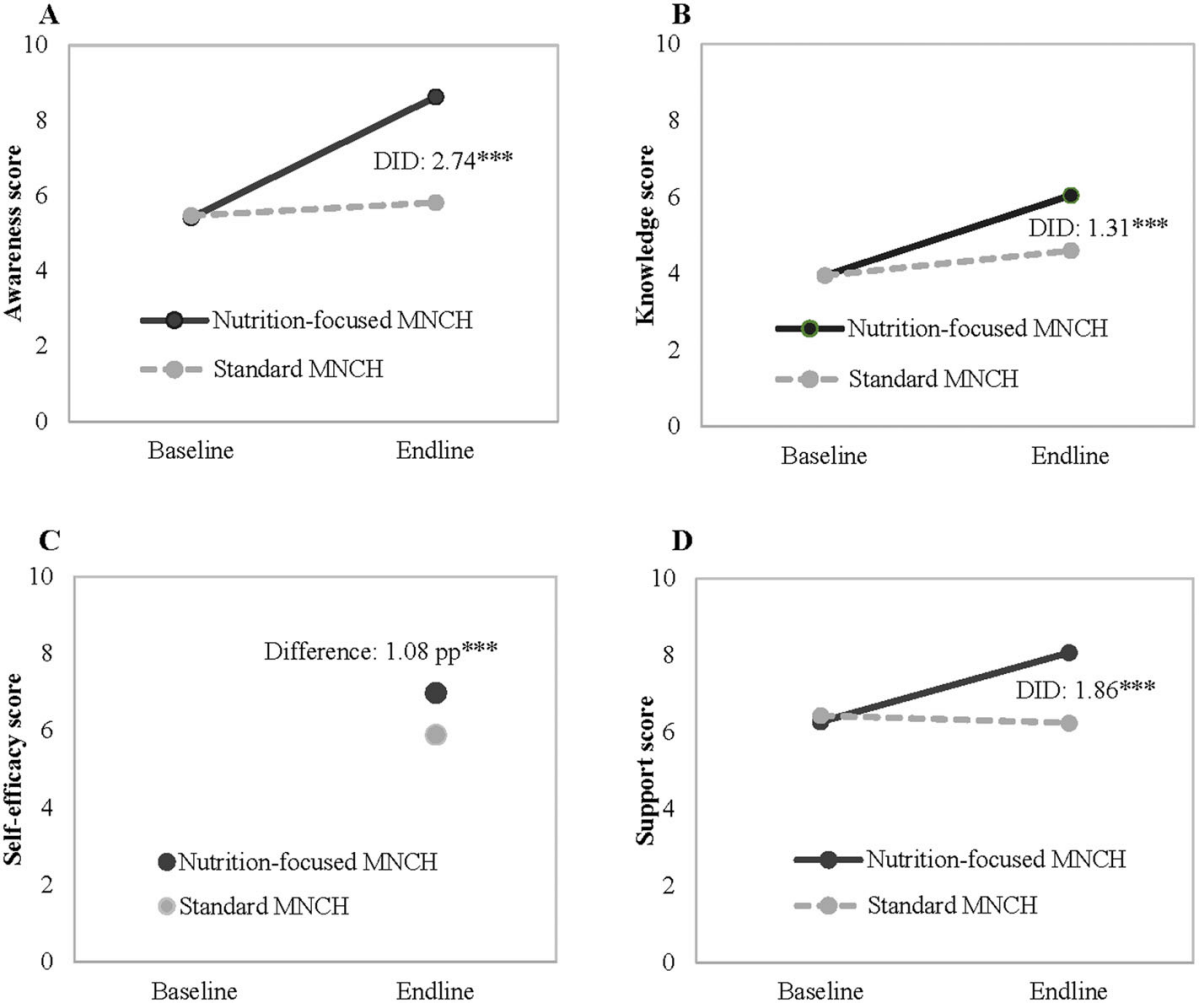
Summary impact of program on husbands’ awareness (A), knowledge (B), self-efficacy (C), and social norms and supports (D). The DID impact estimates from mixed models and 2-tailed *P* values comparing nutrition-focused MNCH and standard MNCH areas in 2014 and 2015, accounting for geographic clustering, are shown. ***P < 0.001. DID, difference in difference; MNCH, Maternal, Neonatal and Child Health; pp, percentage point.

The percentage of husbands who reported ever having heard messages related to nutrition during pregnancy improved over time (**Table 2**) in both groups (for all but 2 messages that did not improve in the standard MNCH group). Improvements were significantly larger in the nutrition-focused MNCH group than in the standard MNCH group. At endline, husbands in the nutrition-focused MNCH group reported significantly higher awareness of specific messages related to proper diet during pregnancy, messages on IFA and calcium, and messages related to weight gain and rest during pregnancy. Overall, the nutrition-focused MNCH group had a 2.74 (95% CI: 2.44, 3.05) greater improvement in husbands’ awareness scores compared with the standard MNCH group (**Figure 2**).

**TABLE 2:** Husbands’ awareness of nutrition message, by program group and survey round^1^

	Baseline		Endline	
Messages	Nutrition-focused MNCH^2^ (*n* = 747)	Standard MNCH (*n* = 767)	Nutrition-focused MNCH^2^ (*n* = 622)	Standard MNCH (*n* = 685)
Proper diet every day during pregnancy ensures weight gain of pregnant woman	77.5	78.5	97.1*	87.7
Proper diet every day during pregnancy ensures adequate growth of baby inside the womb	83.7	82.0	97.3*	90.4
Proper diet every day can ensure quick recovery of mothers after delivery	75.5	72.1	95.3*	86.0
Proper diet every day during pregnancy can save costs on doctor and medicine for both mother and child	46.3	49.3	84.7**	58.1
Proper diet during pregnancy will ensure that the child will be brainy	49.7	53.7	86.0**	61.9
Pregnant women should consume ≥5 different food groups daily	20.6	24.0	83.6***	16.4
Nutritious food is not always expensive	46.9	46.9	75.2***	46.1
During pregnancy, take 1 IFA tablet every day	83.7	78.6	95.8***	84.1
During pregnancy, take 1 calcium tablet every day	84.5*	79.4	96.6***	84.7
During pregnancy, take ≥2 h of rest every afternoon	75.2	70.9	91.6***	72.1
Avoid tea/coffee	14.1	13.0	50.3***	14.9
During pregnancy, a woman should gain 10–12 kg	17.1	26.0	83.6***	21.8
A pregnant woman should be weighed each month	30.5	36.4	84.9***	32.7
Total awareness score (range: 1–10)^3^	5.42 ± 2.12	5.47 ± 2.45	8.63 ± 1.89***	5.82 ± 2.12

^1^Values are means ± SDs or percentages of husbands who had ever heard the messages. *P < 0.05, **P < 0.01, ***P < 0.001. IFA, iron and folic acid; MNCH, Maternal, Neonatal, and Child Health.

^2^Differences between intensive and nonintensive areas, accounting for geographic clustering with the use of a 3-level mixed model.

^3^Unexplained SDs for subdistrict, village, and individual levels were 0.499, 0.285, and 2.02, respectively. Intraclass correlations for subdistrict and village were 0.056 and 0.075, respectively.

#### Husbands’ knowledge related to maternal nutrition

For several measures of husbands’ knowledge on IFA and calcium, the change in knowledge was significantly greater in nutrition-focused MNCH areas than in standard MNCH areas (**Table 3**). Husbands’ knowledge of dietary diversity also improved over time, and the improvement was in favor of nutrition-focused MNCH areas for some specific types of foods, such as thick daal, flesh food, and yellow/orange fruit. Overall, improvements were significantly greater in the nutrition-focused MNCH, compared with standard MNCH, for total knowledge score (DID: 1.31; 95% CI: 1.11, 1.51) (Figure 2).

**TABLE 3:** Husbands’ knowledge of nutrition, by program group and survey round^1^

	Baseline		Endline	
	Nutrition-focused MNCH^2^ (*n* = 747)	Standard MNCH (*n* = 767)	Nutrition-focused MNCH^2^ (*n* = 622)	Standard MNCH (*n* = 685)
Knowledge of IFA				
Coffee or tea decrease iron absorption when taken with meals	3.8	3.5	29.6***	3.5
Benefits of IFA tablets during pregnancy				
To reduce the risk of anemia for pregnant women	34.0	33.1	51.0*	35.2
To reduce the risk of anemia for the child inside the womb	27.6	21.8	48.4**	27.6
To reduce the risk of low birth weight	10.8	11.6	33.8**	14.7
To help improve the child's intelligence	27.0	25.2	36.7	28.0
To reduce the risk of excessive blood loss after delivery	7.8	3.9	21.1***	5.8
To reduce the risk of excessive blood loss during delivery	1.5	2.0	10.8*	3.9
To make the mother healthy/strong	30.3*	40.8	30.1**	45.0
Women should take IFA for 6 mo during pregnancy	29.9	27.3	69.9***	34.3
Knowledge of calcium				
Benefit of calcium tablets during pregnancy				
To ensure adequate growth of child's bones and teeth	31.8	31.0	68.7***	46.1
To reduce the risk of hypertension/pre-eclampsia/eclampsia	6.3	4.4	43.1***	10.7
Women should take calcium for 6 mo during pregnancy	28.1	24.5	69.5***	32.9
Knowledge of dietary diversity				
Kind of food pregnant/lactating women should eat every day				
Rice	75.0	68.5	97.3	99.3
Thick daal	36.1	28.9	83.3***	52.9
Yellow/orange fruit and vegetables	42.7	44.5	73.3	45.4
Dark-green leafy vegetables	80.2	84.5	91.6	84.7
Other vegetables	64.3	65.1	62.1	70.4
Yellow/orange fruit	48.9	55.8	65.6**	43.4
Egg	88.0	87.6	96.6	94.9
Milk/milk products	59.6	70.0	82.8	74.6
Fish/seafood	71.4	71.2	82.2	82.0
Meat (both flesh and organ)	65.9	65.1	85.1*	78.1
Total knowledge score (range: 1–10)^3^	3.95 ± 1.35	3.95 ± 1.45	6.05 ± 1.58***	4.60 ± 1.41

^1^Values are means ± SDs or percentages. *P < 0.05, **P < 0.01, ***P < 0.001. IFA, iron and folic acid; MNCH, Maternal, Neonatal, and Child Health.

^2^Differences between intensive and nonintensive areas, accounting for geographic clustering with the use of a 3-level mixed model.

^3^Unexplained SDs for subdistrict, village, and individual levels were 0.390, 0.259, and 1.31, respectively. Intraclass correlations for subdistrict and village were 0.078 and 0.113, respectively.

#### Husbands’ self-efficacy and social norms related to maternal nutrition

Information on husbands’ self-efficacy and social norms was measured at endline only. Compared with the standard MNCH group, a greater percentage of husbands in the nutrition-focused MNCH group reported a strong positive belief of their ability to support their wives in adopting recommended practices (**Table 4**). Specifically, a higher percentage of husbands in nutrition-focused MNCH areas stated that they could manage to obtain the recommended 5 varieties of food and help their wife in consuming these foods as well as IFA and calcium supplements. In addition, more husbands in the nutrition-focused MNCH group believed that most other husbands in the villages took similar actions to support their wives. The total self-efficacy and social norms score was 1.08 higher in the nutrition-focused MNCH group than in the standard MNCH group at endline (6.99 compared with 5.90) (Figure 2).

**TABLE 4:** Husbands’ self-efficacy and perception of social norms, by program group at endline survey^1^

	Nutrition-focused MNCH^2^ (*n* = 622)	Standard MNCH (*n* = 685)
I can manage to purchase/obtain 5 varieties of food for my wife during pregnancy	93.5***	58.9
I can manage to ensure that my wife consumes adequate amounts of food during pregnancy	93.9***	70.2
I cannot afford to purchase or provide the recommended types and amounts of food every day for my wife during pregnancy because we are poor people^3^	43.2**	62.3
It is too costly to obtain the recommended types and amounts of foods for my wife's consumption during pregnancy^3^	35.3***	57.3
I ensure that there are enough tablets of IFA and calcium at home for my wife to consume ≥180 tablets during pregnancy	92.2***	58.3
I remind my wife to consume all tablets of IFA and calcium as recommended (1/d for 6 mo) during pregnancy	90.9***	53.4
I remind/help my wife to take rest for 2 h during the day in addition to sleeping at night	91.9**	79.8
I review my wife's weight-gain chart and help her find ways to gain enough weight during pregnancy	75.3***	26.2
I know how much weight a pregnant woman should gain during pregnancy	78.0***	29.2
I always call the health worker on my mobile phone if I have any difficulty doing any of the above	81.4***	56.4
Most husbands in my village know the importance of proper nutrition for mother during pregnancy	90.4***	63.9
Most husbands in my village do not purchase diversified nutritious foods and ensure that their wife has these foods available^3^	39.5	49.5
Most husbands in my village remind and encourage their wife to consume the recommended quantity of diversified foods daily	84.8**	62.1
Most husbands in my village know that taking 1 tablet of IFA and 1 tablet of calcium daily for 180 d can prevent their pregnant wife from dying during childbirth and serious complications of pregnancy	95.5***	81.3
Most husbands in my village do not remind/help their wife to take rest for 2 h during the day in addition to sleeping at night^3^	31.4	40.4
Most husbands in my village do not review their wife's weight-gain chart and help her find ways to gain enough weight during pregnancy^3^	38.0	43.8
Most husbands in my village know how much weight a pregnant woman should gain during pregnancy	76.3***	32.8
Most husbands in my village call the health worker on the mobile phone if they have any difficulties doing any of the above	80.7*	52.4
Total self-efficacy and social norm score (range: 1–10)^4^	6.99 ± 1.07***	5.90 ± 0.85

^1^Values are means ± SDs or percentages. *P < 0.05, **P < 0.01, ***P < 0.001. IFA, iron and folic acid; MNCH, Maternal, Neonatal, and Child Health.

^2^Differences between intensive and nonintensive areas, accounting for geographic clustering with the use of a 3-level mixed model.

^3^Reversed coding when creating the score.

^4^Unexplained SDs for subdistrict, village, and individual levels were 0.423, 0.161, and 0.822, respectively. Intraclass correlations for subdistrict and village were 0.203 and 0.232, respectively.

#### Husbands’ support to wives during pregnancy

In nutrition-focused MNCH areas compared to standard MNCH areas, the percentage of wives who reported that their husbands ensured an adequate supply of different kinds of foods and enough tablets of IFA and calcium for them during pregnancy was significantly higher (**Table 5**). Compared with those in the standard MNCH areas, husbands in the nutrition-focused MNCH areas were also more likely to remind their pregnant wives to consume the recommended quantity of diversified foods, take 1 tablet of IFA or calcium daily, and take rest. The overall DID in husbands’ support scores between nutrition-focused MNCH and standard MNCH packages was 1.86 (95% CI: 1.53, 2.19) (Figure 2).

**TABLE 5:** Husbands’ support for following recommended nutrition practices as reported by their wives, by program group and survey round^1^

	Baseline		Endline	
	Nutrition-focused MNCH^2^ (*n* = 747)	Standard MNCH (*n* = 767)	Nutrition-focused MNCH^2^ (*n* = 622)	Standard MNCH (*n* = 685)
Husband purchases diversified nutritious foods and ensures that I have these foods available	69.7	66.6	80.1	73.3
Husband reminds and encourages me to consume the recommended quantity of diversified foods daily	75.5	74.8	89.9**	70.9
Husband helps me to ensure that there are enough tablets of IFA and calcium at home	64.9	62.6	77.2**	55.5
Husband reminds me to take 1 tablet of IFA and 1 tablet of calcium daily	54.6	56.3	73.0***	50.4
Husband remind/helps me to take rest for 2 h during the day in addition to sleeping at night	68.1	66.4	83.9^†^	75.0
Husband and family members do not make me do heavy lifting during pregnancy	84.3	81.2	92.4	89.9
Husband reviews my weight-gain chart and helps me find ways to gain enough weight during pregnancy	21.2**	36.4	69.0***	20.3
Husband calls the health worker on the mobile phone if I have any difficulties to do any of the above	62.8*	70.4	79.9*	64.1
Support scores (range: 0–10)^3^	6.27 ± 2.29	6.43 ± 2.50	8.07 ± 2.23***	6.24 ± 2.37

^1^Values are means ± SDs or percentages. ^†^*P* < 0.10, *P < 0.05, **P < 0.01, ***P < 0.001. IFA, iron and folic acid; MNCH, Maternal, Neonatal, and Child Health.

^2^Differences between intensive and nonintensive areas, accounting for geographic clustering with the use of a 3-level mixed model.

^3^Unexplained SDs for subdistrict, village, and individual levels were 0.472, 0.245, and 2.20, respectively. Intraclass correlations for subdistrict and village were 0.043 and 0.055, respectively.

#### Path analyses for assessment of the role of husbands’ engagement in the program as reflected by behavioral determinants in increasing maternal consumption of micronutrient supplements and dietary diversity

The overall impact of the intervention on IFA and calcium supplement intake and diet diversity has been described elsewhere (32). Briefly, improvements were significantly greater in the nutrition-focused MNCH areas, compared with standard MNCH areas, for the consumption of IFA (DID: 46 tablets), calcium supplements (DID: 50 tablets), and the number of food groups consumed (DID: 1.6 food groups) by pregnant women.

**FIGURE 3:**
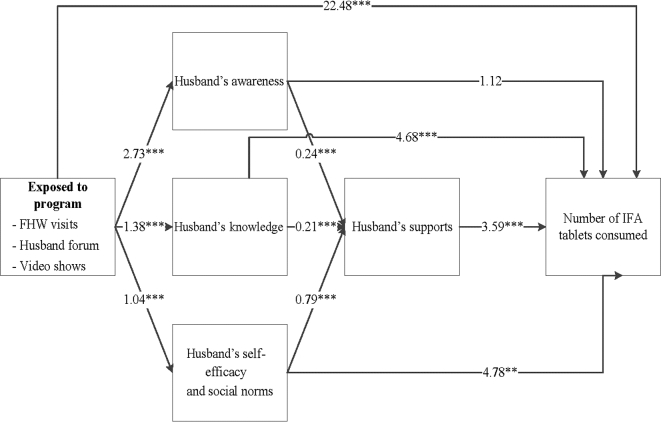
Path analysis for IFA consumption during pregnancy. Values are unstandardized regression coefficients from path analyses. The sum of the indirect differences through husbands’ determinants was 47.9% of the total difference. **P < 0.01, ***P < 0.001. FHW, frontline health worker; IFA, iron and folic acid.

**FIGURE 4:**
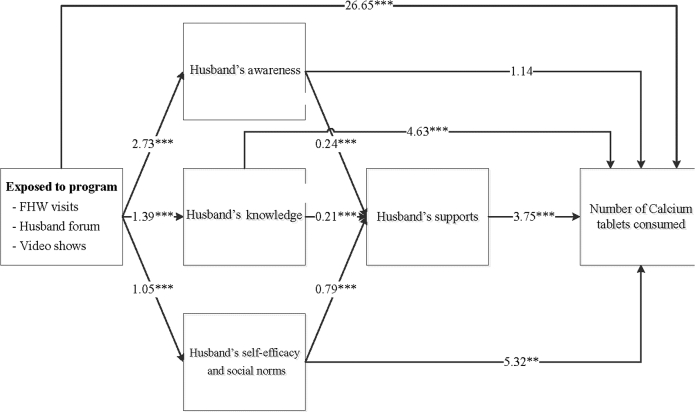
Path analysis for calcium consumption during pregnancy. Values are unstandardized regression coefficients from path analyses. The sum of the indirect differences through husbands’ determinants was 44.6% of the total difference. **P < 0.01, ***P < 0.001. FHW, frontline health worker.

**FIGURE 5:**
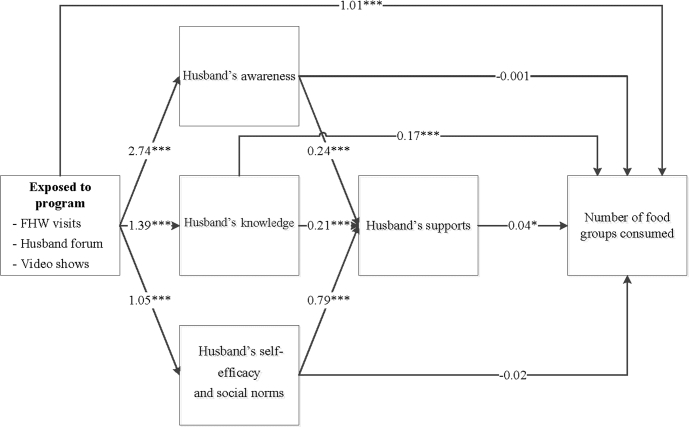
Path analysis for maternal dietary diversity. Values are unstandardized regression coefficients from path analyses. The sum of the indirect differences through husbands’ determinants was 22.0% of the total difference. *P < 0.05, **P < 0.01, ***P < 0.001. FHW, frontline health worker.

The differences at endline in intakes of IFA and calcium supplements and dietary diversity were explained, in part, through improved husbands’ behavioral determinants (**Figures 3****–****5**). The nutrition-focused MNCH program had a significantly higher score on husbands’ awareness (difference of 2.73), knowledge (difference of 1.39), and self-efficacy and perception of social norms (difference of 1.05). These husbands’ behavioral determinants, in turn, were associated with greater husbands’ support (0.21–0.79). Finally, husbands’ support was associated with higher consumption of IFA (Figure 3), calcium (Figure 4), and dietary diversity (Figure 5). Each 1-point difference in husbands’ support (measured on a 1–10 scale) was associated with a greater number of 3.59 IFA tablets, 3.75 calcium tablets, and 0.04 food groups consumed. The indirect differences, obtained by adding the products of the regression coefficients for each path, suggest that for IFA consumption, 48% of the total program difference was explained by improved husbands’ behavioral determinants and supportive activities. These indirect differences were 44% for calcium and 22% for dietary diversity.

## Discussion

Engagement of husbands in a maternal nutrition program significantly improved several behavioral determinants among husbands (such as their awareness, knowledge, self-efficacy, and social norms, and their support to their wives during pregnancy), which, in turn, partially explained the overall program differences that have been previously reported (32). Specifically, husbands’ behavioral determinants explained nearly half of the improvements in women's intake of IFA and calcium during pregnancy and one-quarter of the difference in dietary diversity observed between the standard and the nutrition-focused MNCH programs.

Several features of the program contributed to the significant impact on husbands’ behavioral determinants and supportive behaviors. First, to facilitate husbands’ involvement, the program mobilized several interventions, including inside the home (counseling both husbands and their wives during home visits) and outside the home (husbands' forum and video shows). Husbands’ forums brought together husbands in the community and gave them opportunities to engage in peer-to-peer discussion on issues related to maternal nutrition and how they could support their wives. Second, priority behaviors and “small doable actions” specific to the program context were carefully selected through formative research (unpublished data) to raise husbands’ awareness of better maternal nutrition practices and build their supportive role. For instance, during formative research, poverty emerged as the greatest barrier to following the dietary recommendations, and this prompted discussions on how husbands could purchase low-cost, seasonal fruit and vegetables and locally grown lentils, or gather wild greens more frequently, to support maternal diet diversification. Similarly, to overcome inequitable sex norms, several specific actions were discussed on the ways in which husbands could support their pregnant wives, including doing housework, ensuring adequate supplies of foods and antenatal micronutrient supplements in the home, and reminding and encouraging their wives to eat nutritious food and take adequate rest. The program also reinforced husbands’ perception of community-wide acceptance of the recommended behaviors through visual images, videos, and stories on model husbands. Community opinion leaders who influence male family members were engaged through meetings as well. Men in Bangladesh also usually gather at tea stalls to share information. Although we do not have measures of the potential spillover effects from husbands who attended forums compared with others, it is likely that some key messages reached husbands beyond those who directly participated.

In our study, intervention exposure among husbands was moderate, with two-thirds of husbands participating in ≥1 of the 3 intervention platforms. A study involving husbands in maternal care in Uganda also reported a similar participation rate (65.4%) (39), whereas studies in India reported a lower attendance rate among husbands (35–50%) (18, 40). In rural, resource-poor settings, achieving high participation rates among husbands is difficult because men are typically working away from home during the day. Other barriers to male involvement in maternal health included low levels of knowledge, embarrassment, and social stigma related to the perceived notion that pregnancy and maternity care–related discussions are relevant only to women (15, 40–44). Understanding the specific context is critical to maximizing husbands’ involvement in maternal health. In our study, we did not find an association between husbands’ characteristics (e.g., age, education, occupation) and program exposure. However, intervention exposure among husbands varied across subdistricts, suggesting that implementation may not have been uniform and that additional strategies may have been needed to strengthen implementation.

Although there is ample evidence on the positive effects of male involvement in improving the utilization of prenatal, delivery, and postnatal care services, as well as breastfeeding and child survival (18, 19), few studies have included measurements related to nutrition-specific practices. In a community-based randomized controlled trial in rural Pakistan, education of pregnant women and their husbands on safe motherhood resulted in a higher intake of iron supplements, an improvement of diet, and a lower workload during pregnancy than in the control group (45). With the use of data from 3 rounds of the Demographic and Health Survey, a study in Indonesia reported a significant association between husband support and adherence to IFA supplementation during pregnancy (which was twice that in households in which husbands were supportive) (14). A randomized controlled trial in Kenya also showed a positive impact on calcium adherence among pregnant women who had an adherence partner, 52% of whom were husbands (23, 46). Our study goes beyond the types of analyses presented in previous studies and reports on the evaluation, with the use of a randomized controlled trial, of the impact of a nutrition-focused program targeted to both men and women during pregnancy on a set of husbands’ behavioral determinants. The study also models the contribution of differences in husbands’ behavioral determinants on the adoption of positive nutrition behaviors by women during pregnancy.

Our study found a lower association of husband involvement with dietary diversity than with micronutrient supplement intake. We suspect there are 2 main reasons for this difference. First, micronutrient supplements were provided for free to the women in the nutrition-focused MNCH intervention group and therefore required that women be motivated—and supported by their husbands—to consume them. It appears that the project was successful in achieving these changes. The achievement of greater dietary diversity, on the other hand, is likely to have required more than awareness and motivation on the part of the pregnant women and their husbands and required that families purchase additional foods. The program did not address economic constraints related to acquisition of diverse foods in the low-income setting of our study.

Husbands’ engagement in the nutrition program showed improvement in behavioral determinants, in having a commitment to and a supportive role during pregnancy (14, 23, 42, 46), and in their confidence to overcome economic constraints (15), but there could be concerns about potential unintended consequences. A recent qualitative study suggests that men with high self-efficacy on supporting maternal nutrition may be viewed to be “too involved,” particularly in settings with gendered concepts such as “males as providers” and “females as nurturers” (41). Another concern was related to the perceived power of men over women, especially in regions where sex inequality is a significant barrier to social development and public health (47). To overcome this barrier, several studies suggest creating spaces for men to learn and exchange with other men on supporting maternal and child nutrition to improve their family's health (15, 40–42). Interventions, such as husbands’ forums, therefore become even more relevant to permit such peer-to-peer exchange.

The intervention package was not blinded to implementers or study participants, which could have introduced bias in the outcome assessment, despite the use of a randomized design. Interventions that involve educational outreach make it difficult to blind the providers or the receivers of educational interventions (48). Data for practices covered by the behavior change communication intervention were from self-reports of husbands and their wives, which may raise concerns of social desirability bias. We included 5 control items on awareness (asking about matters not related to the program's promotion) and 4 negative control items on support, but did not find any bias in responses to these questions. In addition, we also assessed for bias that could arise from husbands who were unavailable for the interview in both groups and found no significant differences in the characteristics or outcome measures between women whose husbands were interviewed and women whose husbands were not interviewed (**Supplemental Table 1**). Finally, there was some spillover in that men in standard MNCH areas were exposed to some video shows; this spillover is not a threat to the internal validity of the study because it would bias the impact toward zero.

In conclusion, the maternal nutrition–focused intervention package integrated into an existing MNCH platform and targeted to both wives and husbands significantly improved several husbands’ behavioral determinants and the nutrition support they provided to their wives during pregnancy. These behavioral determinants, in turn, substantially explained the program's impact on women's intake of IFA and calcium supplements and dietary diversity during pregnancy. Targeting both wives and husbands in maternal nutrition programs focused on promoting adoption of optimal nutrition behaviors can contribute substantially to achieve impacts.

## Supplementary Material

Supplement File

## Notes

Supported by the Bill & Melinda Gates Foundation; the Canadian Department of Foreign Affairs, Trade, and Development, through Alive & Thrive, managed by FHI 360; and the Global Affairs Canada (GAC) Research Program on Agriculture for Nutrition and Health (A4NH), led by the International Food Policy Research Institute.

Author disclosures: PHN, EAF, TS, GW, ZM, LMT, BA, KA, SA, MTR, and PM, no conflicts of interest. Alive & Thrive, represented by co-authors TS, ZM, SA, BA, and KA, participated in the study design but not in the data collection or analysis. Alive & Thrive provided specific inputs to the manuscript with regard to intervention design and provided feedback on the interpretation of results. Freedom to publish the study findings was protected contractually in the agreement between the respective funding sources and the International Food Policy Research Institute. All final decisions on the manuscript were made by the researchers.

Supplemental Table 1 is available from the “Supplementary data” link in the online posting of the article and from the same link in the online table of contents at https://academic.oup.com/jn/.

Abbreviations used:

ANC: antenatal care
DID: difference-in-difference
IFA: iron and folic acid
MNCH: Maternal, Neonatal, and Child Health
SK: Shasthya Kormi (salaried health worker)
SS: Shasthya Shebika (community health volunteer worker)

## References

[1] Black RE, Victora CG, Walker SP, Bhutta ZA, Christian P, de Onis M, Ezzati M, Grantham-McGregor S, Katz J, Martorell R Maternal and child undernutrition and overweight in low-income and middle-income countries. Lancet 2013;382:427–51. 23746772 10.1016/S0140-6736(13)60937-X

[2] Black RE, Allen LH, Bhutta ZA, Caulfield LE, de Onis M, Ezzati M, Mathers C, Rivera J; Maternal, Child Undernutrition Study Group Maternal and child undernutrition: global and regional exposures and health consequences. Lancet 2008;371:243–60. 18207566 10.1016/S0140-6736(07)61690-0

[3] Ramakrishnan U, Grant F, Goldenberg T, Zongrone A, Martorell R Effect of women's nutrition before and during early pregnancy on maternal and infant outcomes: a systematic review. Paediatr Perinat Epidemiol 2012;26(Suppl 1):285–301. 22742616 10.1111/j.1365-3016.2012.01281.x

[4] Danaei G, Andrews KG, Sudfeld CR, Fink G, McCoy DC, Peet E, Sania A, Smith Fawzi MC, Ezzati M, Fawzi WW Risk factors for childhood stunting in 137 developing countries: a comparative risk assessment analysis at global, regional, and country levels. PLoS Med 2016;13:e1002164. 27802277 10.1371/journal.pmed.1002164 PMC5089547

[5] Graham W, Woodd S, Byass P, Filippi V, Gon G, Virgo S, Chou D, Hounton S, Lozano R, Pattinson R Diversity and divergence: the dynamic burden of poor maternal health. Lancet 2016;388:2164–75. 27642022 10.1016/S0140-6736(16)31533-1

[6] WHO WHO recommendations on antenatal care for a positive pregnancy experience. Geneva (Switzeland): WHO; 2016. 28079998

[7] Buppasiri P, Lumbiganon P, Thinkhamrop J, Ngamjarus C, Laopaiboon M, Medley N Calcium supplementation (other than for preventing or treating hypertension) for improving pregnancy and infant outcomes. Cochrane Database Syst Rev 2015:CD007079. 25922862 10.1002/14651858.CD007079.pub3 PMC10614032

[8] Haider BA, Bhutta ZA Multiple-micronutrient supplementation for women during pregnancy. Cochrane Database Syst Rev 2015:CD004905. 26522344 10.1002/14651858.CD004905.pub4 PMC6464025

[9] McCauley ME, van den Broek N, Dou L, Othman M Vitamin A supplementation during pregnancy for maternal and newborn outcomes. Cochrane Database Syst Rev 2015:CD008666. 26503498 10.1002/14651858.CD008666.pub3 PMC7173731

[10] Ota E, Mori R, Middleton P, Tobe-Gai R, Mahomed K, Miyazaki C, Bhutta ZA Zinc supplementation for improving pregnancy and infant outcome. Cochrane Database Syst Rev 2015:CD000230. 25927101 10.1002/14651858.CD000230.pub5 PMC7043363

[11] Pena-Rosas JP, De-Regil LM, Garcia-Casal MN, Dowswell T Daily oral iron supplementation during pregnancy. Cochrane Database Syst Rev 2015:CD004736. 26198451 10.1002/14651858.CD004736.pub5 PMC8918165

[12] Imdad A, Bhutta ZA Maternal nutrition and birth outcomes: effect of balanced protein-energy supplementation. Paediatr Perinat Epidemiol 2012;26(Suppl 1):178–90. 22742610 10.1111/j.1365-3016.2012.01308.x

[13] Girard AW, Olude O Nutrition education and counselling provided during pregnancy: effects on maternal, neonatal and child health outcomes. Paediatr Perinat Epidemiol 2012;26(Suppl 1):191–204. 22742611 10.1111/j.1365-3016.2012.01278.x

[14] Wiradnyani LA, Khusun H, Achadi EL, Ocviyanti D, Shankar AH Role of family support and women's knowledge on pregnancy-related risks in adherence to maternal iron-folic acid supplementation in Indonesia. Public Health Nutr 2016;19:2818–28. 27181394 10.1017/S1368980016001002 PMC10270846

[15] Davis J, Luchters S, Holmes W Men and maternal and newborn health: benefits, harms, challenges and potential strategies for engaging men. Melbourne, Australia: Compass: Women's and Children's Health Knowledge Hubs; 2012.

[16] Mullany BC, Becker S, Hindin MJ The impact of including husbands in antenatal health education services on maternal health practices in urban Nepal: results from a randomized controlled trial. Health Educ Res 2007;22:166–76. 16855015 10.1093/her/cyl060

[17] Manjate Cuco RM, Munguambe K, Bique Osman N, Degomme O, Temmerman M, Sidat MM Male partners' involvement in prevention of mother-to-child HIV transmission in sub-Saharan Africa: a systematic review. Sahara J 2015;12:87–105. 26726756 10.1080/17290376.2015.1123643

[18] Varkey LC, Mishra A, Das A, Ottolenghi E, Huntington D, Adamchak S, Khan ME Involving men in maternity care in India. New Delhi, India: Population Council; 2004.

[19] Aguiar C, Jennings L Impact of male partner antenatal accompaniment on perinatal health outcomes in developing countries: a systematic literature review. Matern Child Health J 2015;19:2012–9. 25656727 10.1007/s10995-015-1713-2

[20] Hunter T, Cattelona G Breastfeeding initiation and duration in first-time mothers: exploring the impact of father involvement in the early post-partum period. Health Promot Perspect 2014;4:132–6. 25649998 10.5681/hpp.2014.017 PMC4300437

[21] Shaw A, Golding L, Girard A Alternative approaches to decreasing maternal anemia: identifying the need for social marketing strategies to promote iron‐folic acid supplementation in the Peruvian highlands. Int J Nonprofit Volunt Sect Mark 2012;17(4):325–33.

[22] Tinago CB, Annang Ingram L, Blake CE, Frongillo EA Individual and structural environmental influences on utilization of iron and folic acid supplementation among pregnant women in Harare, Zimbabwe. Matern Child Nutr 2017;13(3). 10.1111/mcn.12350 PMC6866096 27502366

[23] Martin SL, Omotayo MO, Pelto GH, Chapleau GM, Stoltzfus RJ, Dickin KL Adherence-specific social support enhances adherence to calcium supplementation regimens among pregnant women. J Nutr 2017;147(4):688–96. 28250195 10.3945/jn.116.242503

[24] Herba CM, Glover V, Ramchandani PG, Rondon MB Maternal depression and mental health in early childhood: an examination of underlying mechanisms in low-income and middle-income countries. Lancet Psychiatry 2016;3:983–92. 27650772 10.1016/S2215-0366(16)30148-1

[25] Osamor PE, Grady C Women's autonomy in health care decision-making in developing countries: a synthesis of the literature. Int J Womens Health 2016;8:191–202. 27354830 10.2147/IJWH.S105483 PMC4908934

[26] Senarath U, Gunawardena NS Women's autonomy in decision making for health care in South Asia. Asia Pac J Public Health 2009;21:137–43. 19190000 10.1177/1010539509331590

[27] Faruque AS, Ahmed AM, Ahmed T, Islam MM, Hossain MI, Roy SK, Alam N, Kabir I, Sack DA Nutrition: basis for healthy children and mothers in Bangladesh. J Health Popul Nutr 2008;26:325–39. 18831228 10.3329/jhpn.v26i3.1899 PMC2740711

[28] National Institute of Population Research and Training (NIPORT) Mitra and Associates, and ICF International. Bangladesh Demographic and Health Survey 2014. Dhaka, Bangladesh, and Rockville, Maryland, USA: NIPORT, Mitra and Associates, and ICF International; 2016.

[29] Arsenault JE, Yakes EA, Islam MM, Hossain MB, Ahmed T, Hotz C, Lewis B, Rahman AS, Jamil KM, Brown KH Very low adequacy of micronutrient intakes by young children and women in rural Bangladesh is primarily explained by low food intake and limited diversity. J Nutr 2013;143:197–203. 23256144 10.3945/jn.112.169524

[30] Fiedler J, D'Agostino A, Sununtnasuk C Nutrition Technical Brief: A Rapid Initial Assessment of the Distribution and Consumption of Iron–Folic Acid Tablets through Antenatal Care in Bangladesh. Arlington, VA: USAID/Strengthening Partnerships, Results and Innovations in Nutrition Globally (SPRING) Project; 2014.

[31] Nguyen PH, Sanghvi T, Kim SS, Tran LM, Afsana K, Mahmud Z, Aktar B, Menon P Factors influencing maternal nutrition practices in a large scale maternal, newborn and child health program in Bangladesh. PLoS One 2017;12:e0179873. 28692689 10.1371/journal.pone.0179873 PMC5503174

[32] Nguyen PH, Kim SS, Sanghvi T, Mahmud Z, Tran LM, Shabnam S, Aktar B, Haque R, Afsana K, Frongillo EA Integrating nutrition interventions into an existing maternal, neonatal, and child health program increased maternal dietary diversity, micronutrient intake, and exclusive breastfeeding practices in Bangladesh: results of a cluster-randomized program evaluation. J Nutr 2017;147:2326–37. 29021370 10.3945/jn.117.257303 PMC5697969

[33] Fishbein ME, Ajzen I Predicting and changing behavior: the reasoned action approach. New York: Psychology Press; 2009.

[34] Vyas S, Kumaranayake L Constructing socio-economic status indices: how to use principal components analysis. Health Policy Plan 2006;21:459–68. 17030551 10.1093/heapol/czl029

[35] FAO; FHI360 Minimum dietary diversity for women: a guide for measurement. Available from: http://www.fao.org/3/a-i5486e.pdf Rome (Italy): FAO; 2016. Accessed July 30, 2017.

[36] Gertler P, Martinez S, Premand P, Rawlings L, Vermeersch C: Impact evaluation in practice. Washington (DC): World Bank Publications; 2011.

[37] Snijders TAB, Bosker RJ Multilevel analysis: an introduction to basic and advanced multilevel modeling. London: Sage Publishers; 2012.

[38] Kline R Principles and practice of structural equation modeling. 3rd ed. New York: Guilford Press; 2011.

[39] Tweheyo R, Konde-Lule J, Tumwesigye NM, Sekandi JN Male partner attendance of skilled antenatal care in peri-urban Gulu district, Northern Uganda. BMC Pregnancy Childbirth 2010;10:53. 20846369 10.1186/1471-2393-10-53 PMC2946269

[40] Sinha D Empowering communities to make pregnancy safer: an intervention in rural Andhra Pradesh. Health and Population Innovation Fellowship Programme Working Paper No. 5. New Delhi (India): Population Council; 2008.

[41] Catholic Relief Services Father engagement in nutrition: a qualitative analysis in Muhanga and Karongi districts in Rwanda. 2016.

[42] Mullany BC Barriers to and attitudes towards promoting husbands' involvement in maternal health in Katmandu, Nepal. Soc Sci Med 2006;62:2798–809. 16376007 10.1016/j.socscimed.2005.11.013

[43] Kamal MM, Islam MS, Alam MS, Hassan ABME Determinants of male involvement in family planning and reproductive health in Bangladesh. Am J Hum Ecol 2013;2:83–93.

[44] Story WT, Burgard SA, Lori JR, Taleb F, Ali NA, Hoque DM Husbands' involvement in delivery care utilization in rural Bangladesh: a qualitative study. BMC Pregnancy Childbirth 2012;12:28. 22494576 10.1186/1471-2393-12-28 PMC3364886

[45] Midhet F, Becker S Impact of community-based interventions on maternal and neonatal health indicators: results from a community randomized trial in rural Balochistan, Pakistan. Reprod Health 2010;7:30. 21054870 10.1186/1742-4755-7-30 PMC2993657

[46] Martin SL, Omotayo MO, Chapleau GM, Stoltzfus RJ, Birhanu Z, Ortolano SE, Pelto GH, Dickin KL Adherence partners are an acceptable behaviour change strategy to support calcium and iron-folic acid supplementation among pregnant women in Ethiopia and Kenya. Matern Child Nutr 2017;13. 10.1111/mcn.12331 PMC6866070 27507135

[47] Sternberg P, Hubley J Evaluating men's involvement as a strategy in sexual and reproductive health promotion. Health Promot Int 2004;19:389–96. 15306623 10.1093/heapro/dah312

[48] Eldridge S, Ashby D, Bennett C, Wakelin M, Feder G Internal and external validity of cluster randomised trials: systematic review of recent trials. BMJ 2008;336:876–80. 18364360 10.1136/bmj.39517.495764.25 PMC2323095

