# Supplementary material for: Engagement of Husbands in a Maternal Nutrition Program Substantially Contributed to Greater Intake of Micronutrient Supplements and Dietary Diversity during Pregnancy: Results of a Cluster-Randomized Program Evaluation in Bangladesh
Source: J Nutr. 2018 Jun 20;148(8):1352–63. doi: 10.1093/jn/nxy090 (PMC6075465; doi:10.1093/jn/nxy090)
Supplement: Supplement File [file nxy090_supplemental_file.docx]

**Supplemental Table 1: Comparted selected characteristics among women who had husbands interviewed and women without husband’s interviewed^1^**

|  | **Baseline** | | **Endline** | |
| --- | --- | --- | --- | --- |
|  | **Nutrition-focused MNCH** | **Standard MNCH** | **Nutrition-focused MNCH** | **Standard MNCH** |
|  | ***n*=747** | ***n*=767** | ***n*=622** | ***n*=685** |
| **Women with husbands interviewed** |  |  |  |  |
| Program impacts |  |  |  |  |
| Number of IFA tablets consumed | 92.7 ± 63.6 | 92.8 ± 68.7 | 137.2 ± 44.5 | 92.3 ± 63.1***^2^ |
| Number of Calcium tablets consumed | 81.5 ± 61.4 | 80.1 ± 67.5 | 138.5 ± 44.3 | 88.5 ± 63.8*** |
| Number of food groups consumed | 4.53 ± 1.42 | 4.66 ± 1.47 | 5.85 ± 1.63 | 4.46 ± 1.31*** |
| Women’s characteristics |  |  |  |  |
| Age, *y* | 25.0 ± 5.49 | 24.5 ± 5.60 | 25.0 ± 5.57 | 25.6 ± 5.62 |
| Age of married, *y* | 16.7 ± 2.39 | 16.5 ± 2.20 | 16.6 ± 2.41 | 16.8 ± 2.52 |
| Occupation as housewife, *%* | 89.0 | 89.7 | 96.8 | 95.8 |
| Education, *%* |  |  |  |  |
| No schooling | 11.0 | 14.1 | 10.0 | 12.9 |
| Primary school | 37.6 | 32.9 | 30.6 | 36.9* |
| Secondary school | 38.6 | 38.0 | 43.4 | 36.6* |
| High school, college or higher | 12.9 | 14.9 | 16.1 | 13.6 |
| Household characteristics |  |  |  |  |
| Household size, *n* | 5.31 ± 1.93 | 5.09 ± 1.77 | 5.19 ± 1.76 | 5.02 ± 1.60 |
| Number of children < 5 years of age, *n* | 1.34 ± 0.54 | 1.27 ± 0.49* | 1.28 ± 0.49 | 1.22 ± 0.43 |
| Socioeconomic index^3^ | -0.09 ± 0.97 | -0.09 ± 0.93 | 0.14 ± 0.95 | -0.07 ± 0.84* |
| **Women without husbands interviewed** |  |  |  |  |
| Program impacts | 97.4 ± 72.2 | 95.4 ± 71.2 | 140.9 ± 45.9 | 91.6 ± 63.2***^2^ |
| Number of IFA tablets consumed | 85.5 ± 70.0 | 82.8 ± 67.0 | 141.4 ± 45.9 | 85.9 ± 63.2*** |
| Number of Calcium tablets consumed | 4.65 ± 1.46 | 4.70 ± 1.47 | 6.12 ± 1.87 | 4.48 ± 1.29** |
| Number of food groups consumed |  |  |  |  |
| Women’s characteristics | 23.7 ± 5.16 | 23.4 ± 5.43 | 24.3 ± 5.10 | 24.1 ± 5.49 |
| Age, *y* | 16.6 ± 2.57 | 16.5 ± 2.46 | 17.0 ± 2.46 | 16.9 ± 2.42 |
| Age of married, *y* | 90.5 | 92.3 | 95.8 | 93.3 |
| Occupation as housewife, *%* |  |  |  |  |
| Education, *%* | 9.88 | 8.58 | 7.41 | 10.2 |
| No schooling | 32.8 | 37.3 | 32.0 | 26.0 |
| Primary school | 36.0 | 36.9 | 42.1 | 46.0 |
| Secondary school | 21.3 | 17.2 | 18.5 | 17.8 |
| High school, college or higher |  |  |  |  |
| Household characteristics | 4.96 ± 1.88 | 4.63 ± 1.77 | 5.23 ± 2.04 | 5.03 ± 1.66 |
| Household size, *n* | 1.31 ± 0.50 | 1.27 ± 0.47 | 1.37 ± 0.55 | 1.22 ± 0.43** |
| Number of children < 5 years of age, *n* | 0.03 ± 1.02 | 0.03 ± 1.05 | 0.18 ± 1.04 | 0.05 ± 0.84 |
| Socioeconomic index^3^ |  |  |  |  |

^1^Values are means ± SDs or percentages; ^2^Signiﬁcantly different: *p< 0.05, **p< 0.01, ***p< 0.001; ^3^Socioeconomic index was constructed using principal components analysis with variables on ownerships and assets. It is a standardized score with mean=0 and standard deviation=1.

IFA: Iron and folic acid
